# 365. Amoxicillin Direct Oral Challenge in Patients with High-Risk Allergic Reaction

**DOI:** 10.1093/ofid/ofae631.106

**Published:** 2025-01-29

**Authors:** Bryan Le, Shyam Joshi, YoungYoon Ham

**Affiliations:** Oregon State University/Oregon Health & Science University, Clackamas, OR; Oregon Health & Science University, Portland, Oregon; Oregon Health & Science University, Portland, Oregon

## Abstract

**Background:**

Historically, penicillin skin testing (PST) has been the first step in allergy evaluation, followed by an oral challenge (OC). Recently, there have been studies looking at the safety of direct OC in patients without a prior PST, mostly in patients with a history of a mild reaction, such as distant rash or unknown reactions. There is, however, a paucity of data for OC without a PST for patients who report a high-risk reaction to penicillin. Our hospital protocol allows for OC without PST of patients who have had a severe IgE-mediated reaction, as long as the reaction occurred more than 10 years ago.

**Methods:**

Patients over the age of 18 admitted with a high-risk penicillin allergy who were tested with a direct OC were included. High-risk reactions include anaphylaxis, angioedema, hives, as well as shortness of breath. Patients that had a PST prior to an OC or did not have a penicillin allergy were excluded. Data collected were age at the time of challenge, reported allergy and historic reaction, presence of other high-risk allergies, onset to symptoms, time since last reaction, and result of OC. If the challenge resulted in a reaction to penicillin and inability to de-label the allergy, data on reaction and use of rescue medications were also collected.

**Results:**

Of the 288 patients who had received an OC at our institution, 9 were excluded due to prior PST and 2 were excluded due to a non-penicillin allergy. One hundred eight were additionally excluded due to their initial allergic reaction not being high-risk. One hundred sixty-nine patients were included in the analysis. Of the 169 patients with a history of high-risk reactions, 46 patients specifically reported anaphylaxis and 34 patients reported angioedema. One hundred and fifty-nine patients (94.1%) passed the OC and had the allergy de-labeled. Of the 10 patients (5.9%) who reacted during the OC, 3 required diphenhydramine and one required albuterol. The rest did not require the use of rescue medications and were simply observed until the reaction passed. Epinephrine was not used for any patient as a rescue medicine.

**Conclusion:**

Direct OC without a prior PST for patients who report a high-risk allergic reaction to penicillin drugs is reasonable after 10 years have passed from the initial reaction.

**Disclosures:**

**YoungYoon Ham, PharmD**, Gilead: Honoraria

